# Evidence of two mitochondrial lineages and genetic variability in forensically important *Lucilia eximia* (Diptera: Calliphoridae) in Colombia

**DOI:** 10.1093/jme/tjad031

**Published:** 2023-04-18

**Authors:** Juliana Pérez-Pérez, John Alexander Pulgarín Díaz, Andrés López-Rubio, Luz M Gómez-Piñerez, Guillermo Rúa-Uribe, Edna J Márquez

**Affiliations:** Grupo Entomología Médica, Facultad de Medicina, Universidad de Antioquia, Medellín, Colombia; School of Forest Sciences, University of Eastern Finland, Joensuu, Finland; Corporación Colombiana de Investigación Agropecuaria — AGROSAVIA, Centro de Investigación El Nus — Vereda ICA, Corregimiento San José del Nus, municipio de San Roque, Antioquia, Colombia; Grupo Entomología Médica, Facultad de Medicina, Universidad de Antioquia, Medellín, Colombia; Tecnológico de Antioquia I. U., Facultad de Derecho y Ciencias Forenses, Grupo de Investigación Bioforense, Medellín, Colombia; Tecnológico de Antioquia I. U., Facultad de Derecho y Ciencias Forenses, Grupo de Investigación Bioforense, Medellín, Colombia; Grupo Entomología Médica, Facultad de Medicina, Universidad de Antioquia, Medellín, Colombia; Facultad de Ciencias, Escuela de Biociencias, Laboratorio de Biología Molecular y Celular, Universidad Nacional de Colombia Sede Medellín, Medellín, Colombia

**Keywords:** forensic entomology, lineages, *COI*, *Cytb-tRNA-Ser-ND1*, variability, blowflies

## Abstract

*Lucilia eximia* (Wiedemann, 1819) (Diptera: Calliphoridae) is a blowfly with medical and forensic importance that shows genetic and color variation, however, these variations have not justified the description of new species. But in forensic entomology an accurate identification of species and subpopulations is crucial. We explored the genetic variation of *L. eximia* from eight localities, in five natural regions in Colombia using two mitochondrial fragments, including the standard locus for insect identification *COI* and the *Cytb-tRNA-Ser-ND1* region. We found significant differentiation at *COI* and *Cytb-tRNA-Ser-ND1* level, characterizing two lineages and revealing a deep and significant genetic split. High values of *F*_ST_ and genetic distances supported the two lineages. The origin of the divergence of *L. eximia* remains to discover. Examining whether the lineages have diverse ecological and biological behaviors could be a significant impact on the use of *L. eximia* in forensic and medical science. Our results could have relevant implications for the use of post-mortem interval estimation based on insect evidence, as well as our sequences improve the database used in DNA-based methods for identifying forensically important flies.

## Introduction

Many of us relate *Lucilia eximia* (Wiedemann, 1819) (Diptera: Calliphoridae) as an annoying fly, despite its valuable and unexpected uses. Applying controlled myiasis, their larvae remove necrotic tissue of chronic wounds (Wolff et al. 2010). Its developmental rate helps forensic entomologists to estimate the time of death of a corpse (Catts and Goff 1992, Acosta et al. 2021). Also, entomological evidence provides valuable information concerning the circumstances of death, including season, location, movement or storage after death, use of drugs, and even linking child neglect (Benecke and Lessig 2001, Amendt et al. 2007). But the use of entomological evidence relies on accurate species identification and subpopulations (Tomberlin et al. 2011).

Despite its value, morphology-based identification of insects shows limitations (Gemmellaro et al. 2019). Taxonomic keys are not always available and can be difficult to use, especially when identifying immature stages or cryptic species (Harvey et al. 2003, Zehner et al. 2004). *Lucilia eximia* exhibits color patterns and genetic variability when comparing mainland and island specimens (Whitworth 2010, 2014). In one study in Colombia, *L. eximia* exhibited a high level of variability based on the Cytochrome Oxidase I gene (*COI*), suggesting the presence of two species within—also called cryptic species (Solano et al. 2013). However, this study suggested including more specimens from a wider distribution range in Colombia (Solano et al. 2013).

Regarding the color patterns of *L. eximia*, its vestiture varies from silver, silver- gold to gold (Whitworth 2010). Probably this variation is a result of geographical differences. These color variations as well as genetic variation lead to the description of new species in several groups of insects (Guerrero and Fernández 2008, Badejo et al. 2020). Most insects show morphological variation, often related to geographical origin, and landscape alteration—as a result of urbanization (Theodorou 2022). But this variation increases the difficulty to identify (Carolan et al. 2012) and include them in posterior studies.

Mitochondrial genes help to identify insects, including Calliphoridae (Stevens and Wall 2001, Salem et al. 2015, Fuentes-lópez et al. 2020). Specifically, a short fragment of the *COI* gene is reliable for species identification, even identifying cryptic species (Mathur et al. 2012, Scarpassa and Alencar 2012). The Cytochrome b gene (*Cytb*) is also used to study intraspecific variability in Diptera (GilArriortua et al. 2013, Pech-May et al. 2018). For instance, to study the *L. eximia* genetic variability Giraldo et al. (2011) used a fragment of mitochondrial DNA including the *Cytb* and the NADH dehydrogenase genes (*Cytb-tRNA-Ser-ND1*). However, this study showed a moderate level of variability, conversely to the high level of variability revealed by *COI* sequences in Colombian specimens (Solano et al. 2013).

To study the genetic variability of *L. eximia*, we analyzed specimens collected from eight localities in five Colombia natural areas, using two mitochondrial fragments, the *COI* barcode region and *Cytb-tRNA-Ser-ND1.* We expected to find substantial variation between *L. eximia* populations.

## Methods

*Lucilia eximia* adults were collected in eight localities from five natural areas in Colombia defined by IGAC (1997), including highly disturbed areas, semi-rural areas, and secondary forests (Table 1).

**Table 1.** Collection localities for Lucilia eximia specimens

Natural region	Localities, department	Coordinates	*n*
Amazon	Leticia, Amazonas	04°12ʹ40.1″ S 69°56ʹ 32.8″ W	6
Andean	Caldas-Medellín, Antioquia	06°03ʹ06.9″ N 75°37ʹ19.2″ W	9
Andean	Cola del Zorro-Medellín, Antioquia	06°12ʹ19.7″ N 75°32ʹ43.9″ W	9
Andean	Copacabana, Antioquia	06°22ʹ07.1″ N 75°29ʹ22.3″ W	11
Andean	Pajarito-Medellín, Antioquia	06°17ʹ10.7″ N 75°36ʹ43.7″ W	7
Pacific	Playa Huina, Chocó	06°16ʹ0.7″ N 77°27ʹ16.3″ W	3
Caribbean	Santa Marta, Magdalena	11°8ʹ34.20″ N 74°6ʹ31.50″ W	7
Orinoquia	Puerto Gaitán, Meta	04°21ʹ27.96″ N 71°57ʹ00.86″ W	9

We used Van Someren-Rydon traps (DeVries 1987) using a mixture of rotten fish heads and chicken viscera as bait. In each locality, we placed four traps at 1.5 m above the ground for 96 h and separated the traps 200 m from each other. Every 48 h, we replaced the baits and collected the specimens and identified the samples using taxonomic keys for the Neotropical species of *Lucilia* (Whitworth 2010, 2014).

From one specimen leg of the 61 collected specimens, we extracted DNA using the DNeasy Blood & Tissue Handbook (Qiagen) kit. A 650 pb fragment from the *COI* gene was amplified using primers developed by Folmer et al. (1994) and PCR amplification following the Solano et al. (2013) protocols. A second fragment of 492 pb was amplified from the *Cyb-tRNA-Ser-ND1* region, using primers developed by Ready et al. (1997). The PCR products were visualized on 0.8% agarose gel using Ezvision (AMRESCO) under UV light and sequenced bi-directionally (Macrogen Inc. Korea). Sequences were checked against available records in the National Center for Biotechnology Information (NCBI) using the BLAST algorithm. Finally, we verified possible mitochondrial copies at the nucleus (NUMT) by BLASTN search and analyzing codons as suggested by Hlaing et al. (2009) and assembled the sequences using the genome of *L. sericata* (NC_009733) in Geneious v.8.0.4.

We calculated haplotype diversity (Hd), haplotype number (n), nucleotide diversity (π), and genetic diversity parameters using DNASP v. 6.0 (Librado and Rozas 2009) and calculated genetic distances based on Kimura two parameters model. To examine the relationship between haplotypes, we built a haplotype network using Network v. 4.6.2.2. (Bandelt et al. 1999).

Based on the concatenation of the *COI* and the *Cytb*-*tRNA-Ser-ND1* genes, we built a tree using Bayesian Inference Analysis and applied the evolutionary model GTR + T based on the Akaike criterion (Akaike 1974), through MrBayes v. 3.1.2 (Ronquist and Huelsenbeck 2003). We calculated the pairwise *F*_ST_ values using Arlequin v. 3.1 (Excoffier et al. 2005) and used the Mantel test to analyze the correlation between geographic distances and *F*_ST_ values using XLSTAT v.3.9.

## Results

The mitochondrial dataset included 46 sequences, representing 16 haplotypes of the *COI* region (H1 to H16 *COI* haplotypes) and 61 sequences, representing 8 haplotypes of the *Cytb-tRNA-Ser-ND1* region (H1b to H8b*Cytb-tRNA-Ser-ND1* haplotypes). *COI* sequences were deposited in GenBank under accession number KT160173–KT160218 and the *Cytb-tRNA-Ser-ND1* sequences under KU665416–KU665476 (Supplementary S1). Comparing *COI* to *Cytb-RNAt-Ser-ND1*, COI revealed higher values of genetic distances, haplotype, and nucleotide diversity measures. The intraspecific distance for the *COI* region ranged between 0.0% and 6.2% (Table 2).

**Table 2.** Genetic diversity of COI and Cytb-tRNA-Ser-ND1 sequences in Lucilia eximia from Colombia

Molecular marker	Sample	Number of sequences (*n*)	Number of haplotypes (*h*)	Haplotype (frequency)	Haplotype diversity (Hd)	Nucleotide diversity (π)	Genetic distances K2P (%)
COI	Lineage I	17	9	H7(1), H8(1), H9(2), H10(1), H11(3), H12(1), H13(1), H14(6), H15(1)	0.860 ± 0.005	0.005 ± 0.000	0.2–1.2
	Lineage II	29	7	H1(13), H2(8), H3(1), H4(1), H5(1), H6(1), H16(4)	0.724 ± 0.004	0.003 ± 0.000	0.2–2.4
	All samples	46	16	H1(13), H2(8), H3(1), H4(1), H5(1), H6(1), H7(1), H8(1), H9(2), H10(1), H11(3), H12(1), H13(1), H14(6), H15(1), H16(4)	0.873 ± 0.031	0.027 ± 0.002	0.2–6.2
*Cytb-tRNA-Ser-ND1*	Lineage I	19	4	H5b (3), H6b (13), H7b (2), H8b (1)	0.521 ± 0.015	0.002 ± 0.000	0.2–0.6
	Lineage II	42	4	H1b (37), H2b (3), H3b (1), H4b (1)	0.223 ± 0.007	0.001 ± 0.000	0.2–0.6
	All samples	61	8	H1b (37), H2b (3), H3b (1), H4b (1), H5b (3), H6b (13), H7b (2), H8b (1)	0.590 ± 0.062	0.011 ± 0.001	0.2–2.9

H1 to H16 COI haplotypes; H1b to H8b Cytb-tRNAser-ND1 haplotypes of Lucilia eximia. Inside the parentheses is the number of individuals observed for each haplotype.

### Distribution and frequency of haplotypes

**Fig. 1.**
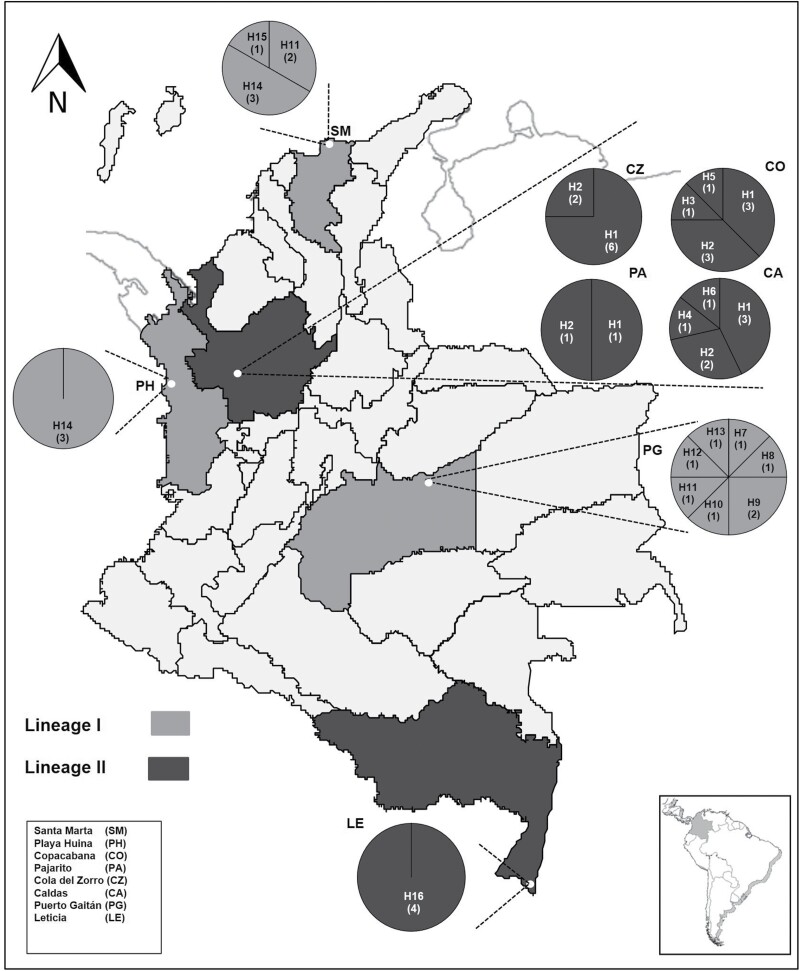
Localities, frequency, and distribution of *COI* haplotypes (H1–H16) of *Lucilia eximia* from Colombia. Numbers in parentheses indicate the number of specimens by haplotype.

We found a total of 16 *COI* haplotypes, their number varied per locality, from one in Playa Huina and Leticia to seven in Puerto Gaitán. H1 (*COI* haplotype1) had the highest frequency (28.3%) followed by H2 (17.4%) both shared among Copacabana, Caldas, Pajarito, and Cola del Zorro, the four closest localities in Antioquia (Fig. 1).

**Fig. 2.**
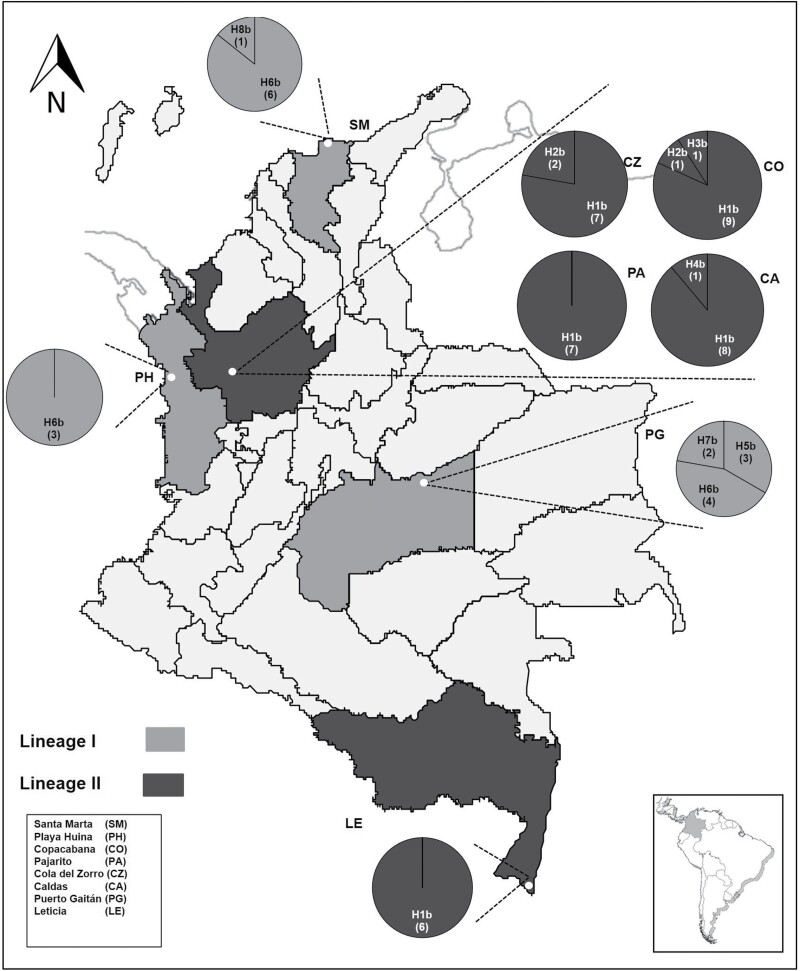
Frequency and distribution of *Cytb-tRNA-Ser-ND1* haplotypes (H1b–H8b) of *Lucilia eximia* from Colombia. Numbers in parentheses indicate the number of specimens by haplotype.

We found a total of 8 *Cytb-RNAt-Ser-ND1*haplotypes, their number varied per locality, from one haplotype in Playa Huina, Pajarito, and Leticia to three in Copacabana and Puerto Gaitán (Fig. 2). H1b (*Cytb-RNAt-Ser-ND1* haplotype 1) was the most frequent haplotype (60.6%) and was found in Cola del Zorro, Copacabana, Caldas, Pajarito, and Leticia—the first four localities are close to each other but far from the last Leticia. The second most frequent haplotype H6b (21.3%) was distributed in Playa Huina, Puerto Gaitán, and Santa Marta.

**Fig. 3.**
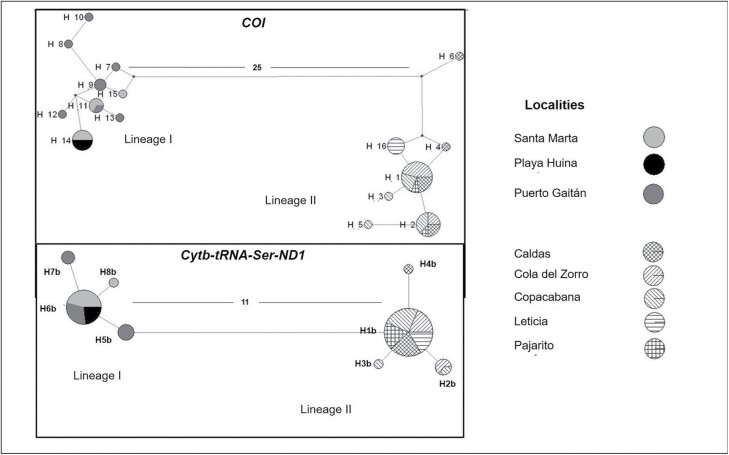
Parsimony haplotypes networks of the *COI* and *Cytb-tRNA-Ser-ND1* genes of *Lucilia eximia* from Colombia. The circle sizes are proportional to the number of haplotypes. Numbers between both groups indicate mutational steps.

Based on haplotype networks *L. eximia* specimens clustered in two groups—hereafter referred to as “lineage I” (*COI*:9 haplotypes; *Cytb-tRNA-Ser-ND1:* 4 haplotypes) and “lineage II” (*COI*: 7 haplotypes; *Cytb-tRNA-Ser-ND1:* 4 haplotypes), separated by a significant number of mutations. The haplotype network based on *COI* showed 25 mutational steps and the haplotype network based on *Cytb-tRNA-Ser-ND1* showed 11 mutational steps (Fig. 3).

### *Lucilia eximia* genetic lineages

**Fig. 4.**
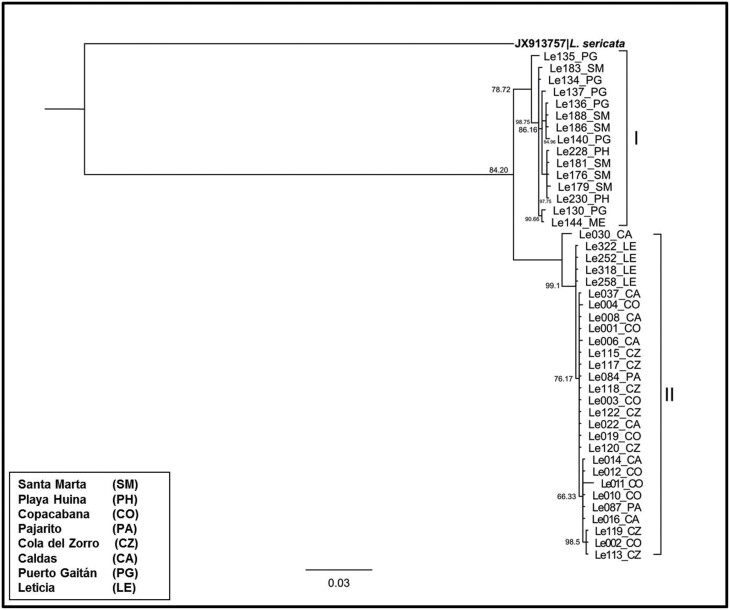
Bayesian Inference topology tree (under the GTR + T model) based on concatenation of COI and Cytb-tRNA-Ser-ND1 genes of Lucilia eximia from Colombia.

The two lineages of *COI* and *Cytb-tRNA-Ser-ND1* showed non-overlapping geographic distribution. The lineage I clustered specimens from Santa Marta, Playa Huina, and Puerto Gaitán, whereas, the lineage II clustered specimens from Caldas, Cola del Zorro, Copacabana, Leticia, and Pajarito. The Bayesian concatenated analysis revealed the same clustering (Fig. 4).

Likewise, the *F*_ST_ values also showed a significant degree of genetic differentiation between the two lineages (COI *F*_ST_ = 0.9334 *P* < 0.001; Cytb-tRNA-Ser-ND1 *F*_ST_ = 0.6685 *P* < 0.001) and the Mantel test indicated the absence of correlation between *F*_ST_ and geographic distances (COI, *r* = 0.0450, *P* = 0.9580 and Cytb-tRNA-Ser-ND1, *r* = 0.2720, *P* = 0.1350). Whereas genetic distance ranged from 0.2% to 6.2% between the two lineages. Lineage I showed higher genetic diversity than the lineage II (Table 2). The genetic distance within the lineage I ranged from 0.2% to 1.2%, while within the lineage II ranged from 0.2% to 2.4%.

For lineage I, Puerto Gaitán exhibited the highest COI haplotype diversity (COI: 7 haplotypes; Cytb-tRNA-Ser-ND1: 3 haplotypes). Specimens from Playa Huina showed the lowest COI and Cytb-tRNA-Ser-ND1 diversity, showing one haplotype. For lineage II, all localities of the department of Antioquia shared the two most frequent COI haplotypes, but the diversity varied per locality. Copacabana showed the highest COI (COI: 4 haplotypes; Cytb-tRNA-Ser-ND1: 3 haplotypes) followed by Caldas (COI: 4 haplotypes; Cytb-tRNA-Ser-ND1: 2 haplotypes), whereas Leticia showed the lowest COI and Cytb-tRNA-Ser-ND1 haplotype diversity with a unique haplotype.

## Discussion

This is the first approach to the genetic variability of *L. eximia* in Colombia using two mitochondrial genes. *Lucilia eximia* showed significant levels of genetic variability with two mitochondrial lineages, revealing a deep and significant genetic split. The mutational steps observed in the haplotype networks supported the idea of *L. eximia* in Colombia could be a cryptic species complex. Hart and Sunday (2007) found strong evidence that DNA sequences from single species typically stick together in a single haplotype network. Changes from these patterns are usually consistent with hybridization or cryptic species diversity (Motoki et al. 2021, Scarpassa et al. 2021).

Studies using the *COI* barcode region for delimiting Calliphoridae showed intraspecific distance values between 0.0%–1.487% in Colombia (Solano et al. 2013) and 0.0%–0.612% in Australia (Nelson et al. 2007). We found genetic distance values higher than these previous reports in Colombia and similar to those reported from the Caribbean Region specimens (Yusseff-Vanegas and Agnarsson 2017). Conversely, as observed in the last study, our findings do not appear to be explained by geographical origin specimens. Our results confirmed a substantial intraspecific variation of *L. eximia* in Colombia using the *COI* barcode and *Cytb-tRNA-Ser-ND1 region*.

The genetic differences we found in *L. eximia* in Colombia may be influenced by the landscape and its characteristics. The idea that anthropization affects the molecular variability of *L. eximia* has not been yet proposed. Ecological conditions, such as the ones resulting from anthropogenic impact, changed the genetic structure of other insects (Ashley et al. 2003, Huber et al. 2008). For instance, two populations of *Aedes aegypti* (Diptera: Culicidae) from the forest and urban areas showed genetic differences attributed to environmental factors (Huber et al. 2008). In our study, we did not choose the localities based on the level of anthropization. However, we noticed that the localities which presented the lineage I showed low anthropization levels (semi-rural areas). While the localities which presented the lineage II were urban areas. We hypothesize that the presence and distribution of the lineages of *L. eximia* may be related to the level of anthropization. However, this explanation, it is beyond our purpose and we need additional research to confirm it.

We detected high levels of genetic variability of *L. eximia* based on mitochondrial regions. However, given the lack of information on the nuclear variability of *L. eximia*, further population studies using nuclear genes are crucial to determine the variation at this level. Studies on other species have shown that mitochondrial genetic variation does not necessarily indicate species differentiation. For example, two populations of the blowfly *Phormia regina* (Diptera: Calliphoridae) showed a substantial level of variation in mitochondrial genes but a low variation in nuclear genes and non-morphological variation. This study concluded that the variation between *P. regina* populations was related to intraspecific mitochondrial divergence rather than species-level differentiation (Jordaens et al. 2013).

Our results could have important implications for the use of *L. eximia* in forensic and other medical fields. In other species, fertility, life cycle, emergence, and adult longevity varied between populations and this variation was related to noteworthy molecular variation (Justiniano et al. 2004, Scarpassa and Alencar 2012, Jordaens et al. 2013). For instance, the forensically important blowfly *P. regina* shows developmental rate variation, which was explained by mitochondrial divergence between populations (Picard and Wells 2009). These findings highlighted the importance of taking into account this variation when using insects in forensic casework. The lineages we found in *L. eximia* may differ biologically, studying the lineages of *L. eximia* in more detail is necessary. Differences in the developmental rate between lineages in *L. eximia* would affect the use of *L. eximia* in forensic entomology applications.

We found significant results when analyzing a small sample of the population, we suggest that future research include more specimens, as well as nuclear genes. In conclusion, *L. eximia* exhibits considerable genetic variability and two mitochondrial lineages in Colombia. It remains to elucidate the origin of this divergence and explore if the lineages exhibit differences in ecological/biological behavior, which may have important implications for the use of *L. eximia* in the forensic field.

## Supplementary Material

tjad031_suppl_Supplementary_Table_S1
