# Supplementary material for: Evidence of two mitochondrial lineages and genetic variability in forensically important Lucilia eximia (Diptera: Calliphoridae) in Colombia
Source: J Med Entomol. 2023 Apr 18;60(4):656–63. doi: 10.1093/jme/tjad031 (PMC10337855; doi:10.1093/jme/tjad031)
Supplement: tjad031_suppl_Supplementary_Table_S1 [file tjad031_suppl_supplementary_table_s1.docx]

**Supplementary Table S1**

| **Lineage** | **Department** | **Locality** | **Sample** | **Sex** | ***COI* Haplotype** | ***Cytb-tRNA-Ser-ND1* haplotype** | ***COI* Accession number GenBank** | ***Cytb-tRNA-Ser-ND1* Accession number GenBank** |
| --- | --- | --- | --- | --- | --- | --- | --- | --- |
| I | Chocó | Playa Huina | Le228 | Female | H14 | H6b | KT160213 | KU665468 |
| I | Chocó | Playa Huina | Le230 | Female | H14 | H6b | KT160214 | KU665469 |
| I | Chocó | Playa Huina | Le232 | Female | - | H6b | - | KU665470 |
| I | Chocó | Playa Huina | Le235 | Male | H14 | - | KT160215 | - |
| I | Magdalena | Santa Marta | Le174 | Female | - | H6b | - | KU665461 |
| I | Magdalena | Santa Marta | Le176 | Female | H14 | H6b | KT160207 | KU665462 |
| I | Magdalena | Santa Marta | Le179 | Male | H14 | H8b | KT160208 | - |
| I | Magdalena | Santa Marta | Le181 | Male | H14 | H6b | KT160209 | KU665464 |
| I | Magdalena | Santa Marta | Le183 | Male | H15 | H6b | KT160210 | KU665465 |
| I | Magdalena | Santa Marta | Le186 | Female | H11 | H6b | KT160211 | KU665466 |
| I | Magdalena | Santa Marta | Le188 | Female | H11 | H6b | KT160212 | KU665467 |
| I | Meta | Puerto Gaitán | Le130 | Female | H7 | H5b | KT160199 | KU665452 |
| I | Meta | Puerto Gaitán | Le131 | Male | H8 | - | KT160200 | - |
| I | Meta | Puerto Gaitán | Le134 | Female | H9 | H6b | KT160201 | KU665453 |
| I | Meta | Puerto Gaitán | Le135 | Male | H10 | H7b | KT160202 | KU665454 |
| I | Meta | Puerto Gaitán | Le136 | Female | H11 | H6b | KT160203 | KU665455 |
| I | Meta | Puerto Gaitán | Le137 | Male | H12 | H6b | KT160204 | KU665456 |
| I | Meta | Puerto Gaitán | Le138 | Female | - | H5b | - | KU665457 |
| I | Meta | Puerto Gaitán | Le140 | Female | H13 | H6b | KT160205 | KU665458 |
| I | Meta | Puerto Gaitán | Le142 | Female | - | H7b | - | KU665459 |
| I | Meta | Puerto Gaitán | Le144 | Female | H9 | H5b | KT160206 | KU665460 |
| II | Amazonas | Leticia | Le252 | Female | H16 | H1b | KT160216 | KU665471 |
| II | Amazonas | Leticia | Le258 | Female | H16 | H1b | KT160217 | KU665472 |
| II | Amazonas | Leticia | Le260 | Female | - | H1b | - | KU665473 |
| II | Amazonas | Leticia | Le306 | Female | - | H1b | - | KU665474 |
| II | Amazonas | Leticia | Le318 | Female | H16 | H1b | KT160218 | KU665475 |
| II | Amazonas | Leticia | Le322 | Female | H16 | H1b | KT160173 | KU665476 |
| II | Antioquia | Caldas | Le006 | Female | H4 | H1b | KT160178 | KU665420 |
| II | Antioquia | Caldas | Le008 | Female | H1 | H1b | KT160179 | KU665421 |
| II | Antioquia | Caldas | Le014 | Female | H2 | H1b | KT160183 | KU665425 |
| II | Antioquia | Caldas | Le016 | Female | H2 | H1b | KT160184 | KU665426 |
| II | Antioquia | Caldas | Le022 | Female | H1 | H1b | KT160186 | KU665430 |
| II | Antioquia | Caldas | Le023 | Male | - | H1b | - | KU665431 |
| II | Antioquia | Caldas | Le024 | Female | - | H1b | - | KU665432 |
| II | Antioquia | Caldas | Le030 | Female | H6 | H4b | KT160187 | KU665434 |
| II | Antioquia | Caldas | Le037 | Male | H1 | H1b | KT160188 | KU665435 |
| II | Antioquia | Cola del Zorro | Le113 | Male | H2 | H2b | KT160191 | KU665443 |
| II | Antioquia | Cola del Zorro | Le115 | Male | H1 | H1b | KT160192 | KU665444 |
| II | Antioquia | Cola del Zorro | Le117 | Male | H1 | H1b | KT160193 | KU665445 |
| II | Antioquia | Cola del Zorro | Le118 | Female | H1 | H1b | KT160194 | KU665446 |
| II | Antioquia | Cola del Zorro | Le119 | Male | H2 | H2b | KT160195 | KU665447 |
| II | Antioquia | Cola del Zorro | Le120 | Female | H1 | H1b | KT160196 | KU665448 |
| II | Antioquia | Cola del Zorro | Le121 | Male | - | H1b | - | KU665448 |
| II | Antioquia | Cola del Zorro | Le122 | Female | H1 | H1b | KT160197 | KU665450 |
| II | Antioquia | Cola del Zorro | Le124 | Female | H1 | - | KT160198 | - |
| II | Antioquia | Cola del Zorro | Le126 | Female | - | H1b | - | KU665451 |
| II | Antioquia | Copacabana | Le001 | Male | H1 | H1b | KT160174 | KU66416 |
| II | Antioquia | Copacabana | Le002 | Female | H2 | H2b | KT160175 | KU665417 |
| II | Antioquia | Copacabana | Le003 | Male | H1 | H1b | KT160176 | KU665418 |
| II | Antioquia | Copacabana | Le004 | Female | H3 | H1b | KT160177 | KU665419 |
| II | Antioquia | Copacabana | Le010 | Female | H2 | H1b | KT160180 | KU665422 |
| II | Antioquia | Copacabana | Le011 | Male | H5 | H1b | KT160181 | KU665423 |
| II | Antioquia | Copacabana | Le012 | Female | H2 | H1b | KT160182 | KU665424 |
| II | Antioquia | Copacabana | Le017 | Male | - | H1b | - | KU665427 |
| II | Antioquia | Copacabana | Le019 | Male | H1 | H1b | KT160185 | KU665428 |
| II | Antioquia | Copacabana | Le020 | Female | - | H1b | - | KU665429 |
| II | Antioquia | Copacabana | Le026 | Female | - | H3b | - | KU665433 |
| II | Antioquia | Pajarito | Le078 | Female | - | H1b | - | KU665436 |
| II | Antioquia | Pajarito | Le080 | Female | - | H1b | - | KU665437 |
| II | Antioquia | Pajarito | Le081 | Male | - | H1b | - | KU665438 |
| II | Antioquia | Pajarito | Le082 | Female | - | H1b | - | KU665439 |
| II | Antioquia | Pajarito | Le084 | Female | H1 | H1b | KT160189 | KU665440 |
| II | Antioquia | Pajarito | Le085 | Male | - | H1b | - | KU665441 |
| II | Antioquia | Pajarito | Le087 | Male | H2 | H1b | KT160190 | KU665442 |

H1 to H16 COI haplotypes; H1b to H8b Cytb-tRNAser-ND1 haplotypes of *Lucilia eximia*.
